# Crosstalk of Multi-Omics Platforms with Plants of Therapeutic Importance

**DOI:** 10.3390/cells10061296

**Published:** 2021-05-23

**Authors:** Deepu Pandita, Anu Pandita, Shabir Hussain Wani, Shaimaa A. M. Abdelmohsen, Haifa A. Alyousef, Ashraf M. M. Abdelbacki, Mohamed A. Al-Yafrasi, Fahed A. Al-Mana, Hosam O. Elansary

**Affiliations:** 1 Government Department of School Education, Jammu 180001, Jammu and Kashmir, India; deepupandita@gmail.com; 2 Vatsalya Clinic, Krishna Nagar, New Delhi 110051, Delhi, India; dt.anunischal46@gmail.com; 3 Mountain Research Centre for Field Crops, Sher-e-Kashmir University of Agricultural Sciences and Technology of Kashmir, Khudwani Anantnag 192101, Jammu and Kashmir, India; shabirhussainwani@gmail.com; 4 Physics Department, Faculty of Science, Princess Nourah bint Abdulrahman University, Riyadh 84428, Saudi Arabia; haalyousef@pnu.edu.sa; 5 Applied Studies and Community Service College, King Saud University, Riyadh 11451, Saudi Arabia; aabdelbacki@ksu.edu.sa; 6 Plant Production Department, College of Food and Agriculture Sciences, King Saud University, Riyadh 11451, Saudi Arabia; mlyafrasi@ksu.edu.sa (M.A.A.-Y.); falman@ksu.edu.sa (F.A.A.-M.); helansary@ksu.edu.sa (H.O.E.)

**Keywords:** medicinal plants, multi-omics platforms, genomics, transcriptomics, proteomics, metabolomics

## Abstract

From time immemorial, humans have exploited plants as a source of food and medicines. The World Health Organization (WHO) has recorded 21,000 plants with medicinal value out of 300,000 species available worldwide. The promising modern “multi-omics” platforms and tools have been proven as functional platforms able to endow us with comprehensive knowledge of the proteome, genome, transcriptome, and metabolome of medicinal plant systems so as to reveal the novel connected genetic (gene) pathways, proteins, regulator sequences and secondary metabolite (molecule) biosynthetic pathways of various drug and protein molecules from a variety of plants with therapeutic significance. This review paper endeavors to abridge the contemporary advancements in research areas of multi-omics and the information involved in decoding its prospective relevance to the utilization of plants with medicinal value in the present global scenario. The crosstalk of medicinal plants with genomics, transcriptomics, proteomics, and metabolomics approaches will be discussed.

## 1. Introduction

### 1.1. Medicinal Plants

The welfare of humankind depends on 12% of Earth’s approximately 300,000 [1,2] to 400,000 plant species. Plant-based herbal medicines have been utilized for more than 5000 years. In the post-Neolithic period, approximately 60% of plants were reported to have medicinal properties [3]. Medicinal plants produce active metabolites or compounds of pharmacological importance for humankind [4]. Payne et al. [5] reported that only 5000 plant species from 250,000–300,000 were thoroughly studied for their medicinal value. Both small, narrow-ranged species and trees have therapeutically vital compounds [6,7]. Traditional medicinal plants are widely exploited in various regions of the world or across different continents, for instance, in South America, Asia and Africa [8], and in diverse civilizations [9,10]. For generations, ethno-medicine has been utilized by around 60% of the world population for their healthcare needs [11]. In prehistoric times when the medicinal properties of herbs were being explored [12,13,14], their scientific relevance, experimental potentiality, molecular mechanisms of medicinal value and emerging omics technologies applicability were unknown. The isolation of the bioactive compound “morphine” from the medicinal plant *Papaver somniferum* L. was the first reported in the early 1800s [15]. Countries such as Korea, China, India and Japan are leading the scientific investigation and validation of the fundamental principles of traditional medicines [16]. The World Health Organization (WHO) has catalogued 21,000 medicinal plants the world over. Out of these, 2500 species are of Indian origin, because of which India is known as the main producer of medicinal plants [17]. The WHO states that up-to 80% of the population of the developing countries depend principally on drugs of plant origin [18,19]. The WHO estimates that plant-based medicines provide principal healthcare for around 3.5–4 billion citizens globally [20]. The International Union for Conservation of Nature and World Wildlife Fund estimated that universally, above 50,000 plant species find use in medicinal purposes [21,22]. Around 25% of pharmaceuticals have a direct or indirect plant-based origin [23,24]. Global Industry Analysts (GIA) hold the opinion that the worldwide plant and herbal supplement market by the year 2020 will be worth USD 115 billion [25]. The WHO reports that the present need of herbal drugs is USD 14 billion per year, and by the year 2050, it will reach to USD 5 trillion [26]. Medicinal plants have been used in Indian therapeutic systems from time immemorial. Indian Medicine Systems include systems of medicine of Indian origin, such as Ayurveda (2559 herbs), Siddha (2267 herbs), Unani (1049 herbs), Sowa-Rigpa (671 herbs), Yoga, Naturopathy and Ethno-botanical Folk (6403 herbs), or those that came to Indian land from exterior areas and became enriched and incorporated into Indian culture, such as homeopathy (460 botanicals), which came to India in the 18th century [27]. The Indian Traditional Medicine System (Ayurveda) is gaining global acceptance. Approximately 25,000 efficient plant-based formulations are exploited within India. The trading of authenticated therapeutic plants and their products universally is worth approximately USD 60 billion. The annual profits of Ayurveda-based medicines in the global market are around USD 813 million [28]. Consequently, the Indian market is the heart of therapeutic plant trading, with a computable trade of around USD 140 million per annum. In 2010, the international export of plant-based and natural ingredients was worth around USD 33 billion and was anticipated to reach USD 93 billion by the year 2015, whereas the export of Indian medicinal plants and their products was predicted to be USD ~0.2 billion. Besides the global business, the worldwide trade of medicinal plants in India generates revenue of USD 1.6–1.8 billion [29]. India contributes only 2.5% of the annual USD 60 billion that constitutes the total worldwide herbal market. In spite of the rich legacy of Ayurvedic literature and the huge biodiversity of medicinal plants, India is still fighting with the potential market demands [29]. Herbaceous plant species (86 genera of 29 families) of Jammu province [30] and 105 medicinal plant species (36 families) from diverse niches of the district of Samba in the Union Territory of Jammu and Kashmir are used for medicinal purposes [31]. There are about 41 dicotyledonous plant species (29 families) [32] and 13 monocotyledonous angiospermic plant species (of 5 families) in the Union Territory of Jammu and Kashmir which are useful in the healing of diabetes mellitus [33].

### 1.2. Omics Technologies

Modern day omics approaches, which include genomics, transcriptomics, proteomics and metabolomics, are becoming extremely significant for the identification and characterization of vital gene-protein-metabolite networks, new drug metabolites, complete genomes, transcriptomes, and proteomes of medicinal plants, and the responses of human cells to drugs or whole ethno-botanical plants for the medicinal use and large-scale production of plant-derived medicines [34,35,36,37]. The next generation sequencing (NGS) technique has increased the deep transcriptome studies of medicinal plants, the crosstalk between gene expression co-responses and the accumulation of metabolites. The concept of “guilt-by-association” states that genes undergoing co-expression lead to biosynthesis of metabolites which show accumulation analogous to levels of the co-expression of genes [38]. The metabolomics approach, along with the functional genomics of gene products from ethno-medicinal plants, accelerates the discovery of new biosynthetic pathways of various bioactive metabolites. This has improved the prospective discovery and generation of products with pharmaceutical significance. Artemisinin (antimalarial drug) was first enhanced by the breeding of *Artemisia annua* L. with novel hybrids with high-yielding abilities so as to gain a robust cropping system, and secondly in a re-engineered microbial host by the reconstitution of the biosynthetic pathway of artemisinin, which was obtained after the sequencing of its genome [39]. The crosstalk of omics with the ethno-botanical approach was studied in a non-plant bioengineered host via the detection of a string of FAD2 phytochemicals after the transcriptomics and metabolomics of developing seeds that amass abnormal fatty acids [40]. *Vitis vinifera* L. multi-omics (transcriptomics, metabolomics and genome-wide microarray analysis) identified 238 genes and 2012 metabolites that are upregulated by UV-C irradiation. This supports the concept that stilbene biosynthesis encourages transcription factor-mediated regulation [41]. Genes of the morphine biosynthetic pathway in *Papaver somniferum* L. have been identified mostly by omics approaches [42,43]. The integration of transcriptome and genetic approaches identified an alkaloid biosynthesis gene cluster in the genome of *Papaver somniferum* L. [44]. In an editorial by Chen [45], phytochemicals such as carotenoids, flavonoids, lignans, and phenolic acids were reported as having been analyzed via high-throughput tools. The mechanistic insights into the bioactivities of these substances, and roles in disease treatments, profiling, extraction, identification and biotechnology, and focus on the gene transfer and nanoparticles have been explored. Another paper focused on studies of medicinal plants with bioinformatics-assisted tools. Their focus was a case study of multi-omics data-based workflow for the Dendrobium medicinal plant, and it incorporated very few details on the genomics, transcriptomics, proteomics and metabolomics of other important medicinal plants [46]. The potential applications of metabolomics as well as analytical techniques, statistical approaches and bioinformatics tools help us to understand the system-wide effects of Thai traditional medicine [47]. The frequency and distribution of the 47,700 microsatellites or simple sequence repeats (SSRs) from 109,609 expressed sequence tags (ESTs) of 11 medicinal plants with antidiabetic potential were studied for their potential as biomarkers for cross transferability [48]. Our present review is novel in terms of being comprehensive and detailed, focusing mainly on the studies of multi-omics technologies in medicinal plants. The review also highlights the chemical structures of medicinal metabolites and/or drugs/synthetic derivatives of some important medicinal plants, along with their medicinal properties. The objective of this review paper is to merge the omics-based approaches with the scientific investigations in various medicinal plants with representative case studies. This will update our awareness of natural-product-based drugs from various plants of therapeutic significance, and the prolonged exploitation of plant pharmaceutical resources.

## 2. Omics in Medicinal Plants

**Figure 1:**
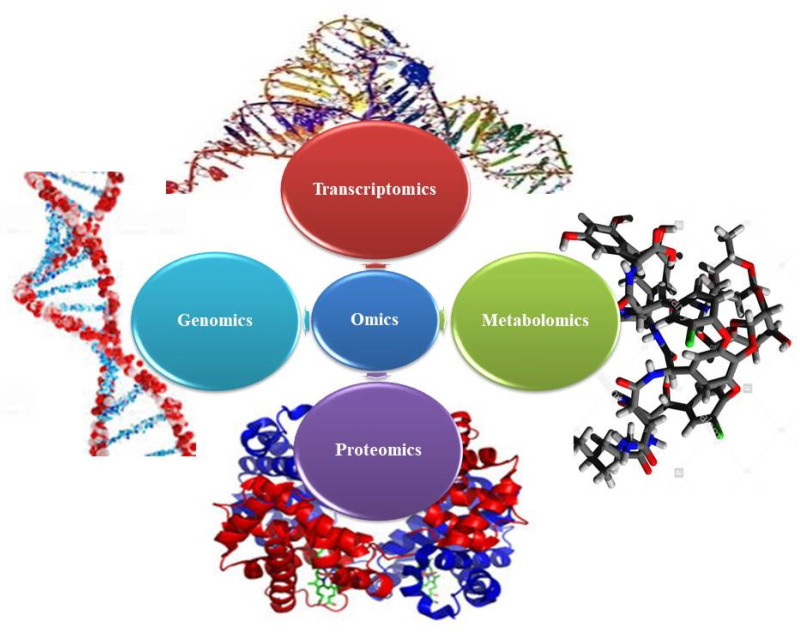
Omics in Medicinal Plants.

High-throughput omics platforms such as genomics, transcriptomics, proteomics and metabolomics (Figure 1) generate big data. Big data can be used for the prediction of secondary metabolic pathways of various therapeutic plants, to discover the genes involved in the biosynthesis of biologically active metabolites and to probe the plant genome and evolution. Medicinal plants develop novel traits to adapt to shifting environments for an improved life. The hypothesis-based and big data-based investigations of medicinal plants combine plant-based analysis, biotechnology and omics approaches to improve the life of man. The Medicinal Plant Genomics Consortium and genome-guided research [49,50], the Medicinal Plant Transcriptome Project [51], the 1000 Green Plant Transcriptome Project [52], etc., will help in the identification of various plant biosynthetic pathways and their evolution, especially in the discovery of new pathways originating as gene clusters in *Oryza sativa* L., barley [53] and poppy. In poppy, an antitumor noscapine (alkaloid) biosynthetic pathway of a 10-gene cluster positioned over 401 kb of genomic sequence was found [44]. Advanced RNA sequencing techniques enable us to study the expression profiles of enzymes and transcription factors on a global scale. The database of metabolomics and transcriptomics of 14 medicinal plants (http://metnetdb.org/mpmr_public/ accessed on 17 November 2020) is accessible for the development of a hypothesis regarding the role of genes [54]. The research area of metabolomics was developed after genomics, transcriptomics and proteomics, and deals with all the metabolites of a cell [55]. In *Glycyrrhiza uralensis* Fisch. ex DC. (licorice), two cytochrome P450 genes of the glycyrrhizin biosynthetic pathway were detected [56], and these direct the microbial generation of glycyrrhetinic acid and triterpene saponin, which is a natural sweetener.

## 3. Genomics in Medicinal Plants

The DNA sequences of a genome integrate vital information of the origin, development and epigenomic regulation of a plant. This may act as the foundation of decoding genomic and chemical diversity at minute levels [57]. The high-throughput sequencing of various therapeutic plants emphasizes the biosynthetic pathways of their drug molecules, secondary metabolites [58], and regulatory pathways. The genomic sequencing of plants helps us to investigate various genes and regulatory sequences of medicinal importance. Whole-genome sequencing, besides being a costly process, is also demanding when the genome contains a huge share of repetitive sequences, elevated levels of heterozygosity, and non-diploids [59]. The sequenced genome of grapes is available online (http://www.genoscope.cns.fr/spip/ accessed on 17 November 2020) [60,61]. *Phalaenopsis equestris* (Schauer) Rchb.f [62], *Brassica napus* L. [63], *Capsicum annuum* L. [64,65], *Momordica charantia* L. [66], *Coffea canephora* Pierre ex A. Froehner [67], *Salvia miltiorrhiza* Bunge [68], *Ziziphus jujuba* Mill. [69,70], *Glycyrrhiza uralensis* Fisch. Ex DC. [71], *Dendrobium officinale* Kimura et Migo [72], *Azadirachta indica* A. Juss., 1830 [73], and *Catharanthus roseus* (L.) G. Don chloroplast and genome [49,74] and the chloroplast of *Pogostemon cablin* (Blanco) Benth. [75] have been sequenced. These may emerge as significant models for the study of herb genetics and their cell metabolic actions [49,76]. *Salvia miltiorrhiza* Bunge (Danshen) and *Catharanthus roseus* (L.) G. Don synthesizes triterpenes, indole alkaloids and diterpene quinone. The draft genome sequence of *Catharanthus roseus* (L.) G. Don offers verification of the partial gene clustering for alkaloid (vinblastine and vincristine) biosynthesis. With bacterial artificial chromosome sequencing, Kellner et al. [49] showed seven small clusters, each one of two to three genes, in the biosynthetic pathways of vinblastine and vincristine. *Ziziphus jujuba* Mill. is rich in vitamin C and sugar, and has a variety of medicinally essential flavonoids, phenolics and alkaloids. *Azadirachta indica* A. Juss. is medicinally effective as an antitumorigenic, antidiabetic and antimalarial. The genome of neem sequenced with the next generation sequencing approach is loaded with AT sequences and modest repetitive DNA, and has 20,000 genes [77]. The genome-wide identification of *Salvia miltiorrhiza* Burge (Danshen) and the characterization of its putative genes involved in the terpenoid biosynthetic pathway was studied by Ma et al. [78]. The cp genome draft sequence of *Salvia miltiorrhiza* Burge (Danshen) [79] verifies its genome size as approximately 600 MB, having 30,478 genes that code for protein and 1620 genes that act as transcription factors. Some of these take part in the biosynthetic pathways of tanshinone and phenolic acids, which are useful in the treatment of hyperlipidemia, and cardiovascular and cerebrovascular diseases. In *Salvia miltiorrhiza* Burge (Danshen), 40 terpenoid biosynthetic pathway genes have been discovered. Among these, 27 genes were new, which comprise 19 families (10 with single and 9 with multigene). In the terpenoid biosynthetic pathway of *S. miltiorrhiza* Burge (Danshen), *HDR*, *DXS*, *HMGR* and *GGPPS* enzymes are transcribed by multigene families that have diverse expression profiles and subcellular positions [78]. The domestication and differentiation of Capsicum was undertaken by the comparative genome sequencing of cultivated *Capsicum annuum* pepper variety Zunla-1 with wild progenitor *Capsicum annuum* var. glabriusculum (Chiltepin) [64]. The genome sequences of *Solanum tuberosum* L., Capsicum, Atropa and tomato give information about the evolution of various members of the Solanaceae family. Innumerable anticancerous plant-based chemical molecules, such as camptothecin and podophyllotoxin derivatives [80], can be probed with this strategy. Modern day next generation sequencing of the *Panax ginseng* cp genome gives an insight into its evolution and polymorphism [81]. *Panax ginseng* promotes health and is useful in clinical therapy. The genome sequencing of *Dendrobium officinale* Kimura et Migo, which is a medicinal plant, and the orchid Cypripedium macranthos Sw. provides an insight into the content and order of their genes and latent RNA editing sites [82]. *Ocimum sanctum* L. and *Ocimum basilicum* L. genome sequencing and annotation reveals higher expressions of genes of the phenylpropanoid/terpenoid biosynthetic pathway, cytochrome P450s and transcription factors. This has provided a new approach for the mining of biosynthetic pathways of important medicinal metabolites in related species [83,84]. The cp genome sequence of *Ocimum tenuiflorum* L. disclosed that the amino acid mutations at the gene loci of biosynthesis give it incomparable pharmaceutical traits. *Ocimum tenuiflorum* L. generates specialized metabolites with anticancer potential, such as ursolic acid, oleanolic acid, luteolin, taxol, eugenol, apigenin and sitosterol, as a defense mechanism. The genes responsible for the expression of these metabolites with anticancer potential can be identified and used for the development of targeted drugs [85]. In opium poppy (*Papaver somniferum* L.), the genes of benzylisoquinoline alkaloid (BIA) biosynthesis have been identified by virus-induced gene silencing (VIGS) technology. This has also helped us to discover the genes of the morphine biosynthetic pathway involved in reactions of O-demethylation with metabolite thebaine to produce codeine and then the conversion of the metabolite codeine into morphine [42,43]. The genomic data from restriction site-associated DNA sequencing (RAD-Seq) [86], which is used to assess the genome heterozygosity, and genotyping by sequencing (GBS) method are employed to establish the origin and distribution blueprint of the plants with anticancer potential [57]. Microsatellite markers assist in the reproduction of plants, the regulation of the genome, recombination, quantitative genetic variation, evaluation and organization, evolution, and the defense of the genetic resources. In the hemi-parasitic plant *Viscum coloratum* (Kom.) Nakai, with anticancerous properties, 19 new polymorphic microsatellite markers were developed, so that the ecological conservation and population genetics of this plant can be studied [87]. The study of evolutionary genomics helps in identifying genes involved in specific innovations, botanical diversity, as well as medicinally important characteristics, for example, anti-allergic, anticancerous, anti-inflammatory, etc. [57]. ISSR analysis in 32 native populations of an endangered Berberidaceae medicinal plant *Sinopodophyllum hexandrum* (Royle) Ying showed its genetic diversity and structure of population, and provided data for the studies of evolution and conservation [88]. The genome resources assist in the association of genomic variations with the origin of new phytochemicals and physiological traits in the therapeutic plants [89]. To conclude, the genomics of medicinal plants provide information on the genes and regulatory sequences, latent RNA editing sites, origin, evolution, development, domestication, differentiation, polymorphisms, epigenomic regulation, genome heterozygosity, genotyping and biosynthetic pathways of drug molecules, secondary metabolites and their regulatory pathways. However, it is a costly and demanding process in genomes with higher percentages of repetitive sequences and heterozygosity.

## 4. Transcriptomics in Medicinal Plants

The transcriptomes of hundreds of therapeutic plants for instance, Oenothera (Onagraceae) [90], Fabaceae [91], Caryophyllales [92], *Polygonum cuspidatum* Sieb. et Zucc. [93], *Rhodiola algida* (Crassulaceae) [94], *Taxus mairei* [95], *Salvia sclarea* L. (Lamiaceae) [96], etc., subjected to sequencing are available online at the Sequence Read Archive (SRA), National Centre for Biotechnology Information (NCBI), PubMed and Gene Expression Omnibus (GEO) databases. High-throughput comparative transcriptomics of medicinal plants is more viable in comparison to comparative genomics. Transcriptomics is an efficient approach to retrieve the genomic data from numerous non-model therapeutic plants that lack a reference genome. The transcriptomic studies help in the characterization of key characteristics involved in the formation of secondary metabolites and in probing pharmaceutically important mechanisms at the molecular level [94,95,96,97]. With RNA sequencing (RNA-seq), gene sequences can be obtained from plants without a reference genome, and with them integrated analyses of transcriptomics (transcriptome data) and metabolomics (metabolic profiling data sets) potential for any medicinal plant [98]. Whole transcriptome shotgun sequencing (WTSS) makes it possible to probe the genes of various metabolite biosynthesis processes and the relationship between the genes and plant metabolites.

From transcriptome data of *Podophyllum hexandrum* Royle, candidate genes were selected and combinatorially expressed in *Nicotiana benthamiana* Domin. This way, six enzymes from the podophyllotoxin biosynthetic pathway to etoposide aglycone were identified [99]. Podophyllotoxin is a natural precursor of etoposide, which is a chemotherapeutic anticancerous molecule. The podophyllotoxin biosynthetic pathway is only partly known [99]. Simultaneous co-expressions of 10 genes in model plant tobacco led to the reconstitution of the biosynthetic pathway into etoposide aglycone. The etoposide aglycone is a natural lignin and mediator precursor of anticancerous etoposide. The olivetolic acid cyclase enzyme, a polyketide synthase and an acyl-activating enzyme that is responsible for the synthesis of olivetolic acid, was identified from the transcriptome data of glandular trichomes of the female flower of cannabis (*Cannabis sativa* L.) Glandular trichomes of the female flower of cannabis are the primary sites of cannabinoid biosynthesis, suggesting their unnoticed role in the generation of chemical diversity [100,101]. *Chlorophytum borivilianum* Santapau & R. R. Fern. has revealed antitumerogenic and anticancerous potential [102] due to chloromaloside-A, spirostanol-pentaglycosides-embracing beta-D-apiofuranose and steroidal glycosides. The medicinal phytometabolites, such as saponins, alkaloids, terpenoids and polysaccharides, some of which are antitumerogenic, have been identified in Ranunculaceae. The expression profiling of various genes and appropriate transcriptomics platforms have revealed the distinct outcomes of phyto-metabolites in cancerous cells [103]. The transcriptomic data of *Catharanthus roseus* (L.) G. Don generates diverse iridoid-based monoterpene indole alkaloids along with anti-cancerous vinblastine [104]. From these transcriptomic data, a new iridoid synthase was identified that converts 10-oxogeranial into iridoid scaffold [105] and cytochrome P450 hydroxylating genes concerned with the monoterpenoid indole alkaloid biosynthetic pathway [76]. Iridoids possess anti-inflammatory, anticancerous and antibacterial potential [106,107]. Curcuma longa L. decreases the prevalence of gastrointestinal cancers due to secondary metabolite Curcumin. *Curcuma longa* L. (rhizome) transcriptomics showed transcripts associated with the terpenoid biosynthetic pathways and other biosynthetic pathways of various anticancerous phytochemicals, such as vinblastine, curcumin and taxol. This information paved the way for the biosynthetic pathways of a variety of terpenoids in *Curcuma longa* L., along with its transcriptomic database [108]. Phylotranscriptomic approaches provide knowledge about the evolution of the essential anticancerous traits of a plant attributed to its inestimable chemo diversity [109]. Rubiaceae member, *Ophiorrhiza pumila* Champ. ex Benth., accrues a monoterpenoid indole alkaloid known as camptothecin, which is an anticancerous metabolite. The deep transcriptome analysis of *Ophiorrhiza pumila* Champ. ex Benth. yielded a 2GB sequence from which novel genes of plant secondary metabolic biosynthetic pathways were predicted [98]. *Withania somnifera* (L.) Dunal synthesizes bioactive secondary metabolites known as withanolides. The leaf and root tissue chemo-profiling of *Withania somnifera* (L.) Dunal chemovars showed variations in the composition and traits of withanolides. The genes of chemotypes and the tissue-specific biosynthesis of withanolide of distinct chemotypes was characterized [110]. The differential transcriptomics and metabolic profiling of engineered culture cells that do or do not produce camptothecin alkaloids identifies candidate genes of the biosynthesis of alkaloids and anthraquinones [98,111]. The combination of transcriptomics and genetic approaches proposes the existence of a complex gene cluster in the genome used for alkaloid noscapine biosynthesis in the chemo-variety of *Papaver somniferum* L. [44]. Advanced genome sequencing and comparative transcriptomics have advanced our understanding of the functions of diverse genes involved in the biosynthetic pathways. By cDNA microarray from various stages of hairy root development in *Salvia miltiorrhiza* Burge (Danshen), Cui et al. [112] found variations in the expression profiles of the genes of the tanshinone biosynthetic pathway. From RNA-seq data of *Salvia miltiorrhiza* Burge (Danshen), a group of novel genes related to terpenoid-derived tanshinone and salvianolic acid secondary metabolite biosynthesis was identified [113]. In the five key secondary metabolic biosynthetic pathways that cover almost all bases in the phenylpropanoid and terpenoid pathways, the identification of 1,539 unigenes was possible. The functional characteristics of approximately 70 new transcripts of phenylpropanoid and terpenoid biosynthetic pathways and the spatiotemporal expression profiles of 10 novel transcripts correlated to terpenoid and phenolic acid biosynthetic pathways were understood through the transcriptome. The differential gene expression profiling of *Lupinus angustifolius* L. chemo-varieties for quinolizidine alkaloids, the gene that encodes the enzyme lysine decarboxylase involved in the first step of the alkaloid biosynthetic pathway, was undertaken, and the catalytic potential of the lysine decarboxylase was explained by site-directed mutagenesis and protein modeling [114]. The putative homologue genes of the iridoid biosynthetic pathway were identified from the transcriptome of a variety of TIA-producing therapeutic plants [115]. Then, they were evaluated with non-secologaninous plants to remove unrelated genes, and the authors validated the necessity of CrDL7H- 7-deoxyloganic acid 7-hydroxylase (CYP72A224) in iridoid metabolism and its homologous nature with the secologanin synthase-like gene of *Camptotheca acuminata* Decne. [116]. RNA-seq gives rich sequence information about the full-length gene sequences, and also helped recognize the orthologous and paralogous gene cluster families in *Camptotheca acuminata* Decne., *Rauvolfia serpentina* (L.) Benth. ex Kurz and *Catharanthus roseus* (L.) G. Don for the respective biosynthesis of camptothecin, ajmaline and vinblastine [115,117]. In 2012, Gongora-Castillo and coworkers developed a transcriptomics database. From this database, the alcohol dehydrogenase homologue known as the tetrahydroalstonine synthase (THAS) gene, which was upregulated by MeJA and converts strictosidine aglycone into tetrahydro-alstonine, was validated via VIGS, nuclear magnetic resonance (NMR) imaging and liquid chromatography–mass spectrometry (LC-MS) [118]. The RNA-seq of two *Vaccinium macrocarpon* Aiton (Cranberry) fruits at different developmental stages was annotated from public domains such as NCBI, KEGG, GO and NT, and the genes *CHS*, *F3H*, *CHI*, *F3* ′ *H*, and *LDOX* of the bioactive flavonoid biosynthetic pathway were identified [101]. The cranberry transcriptome, in comparison to blueberry (Vaccinium sp.), reveals a *UDP*-*glucose flavonoid 3-O-glucosyl transferase* (*UFGT*) enzyme of the flavonoid biosynthetic pathway, which characterizes more different types of flavonoids that are accessible in the cranberry transcriptome. Further, the ABC transporters and glutathione S-transferases (GST), WD40, WRKY and bHLH regulatory transcription factors, involved in flavonoid biosynthesis were also found in this transcriptome [119]. The metabolic changes that take place in the fruit-ripening of *Rubus coreanus* Miq. 1867 (rich in anthocyanins) were assessed by metabolomics and transcriptomics [120]. From the data of transcriptome, the annotated unigenes of flavonoid metabolism, along with main genes such as F3H, CHS and CHI, were identified. In rice, tomato and petunia, the CHI gene is the chief gene of the flavonoid and anthocyanin biosynthetic pathway [121,122,123]. The CHI enzyme family was screened from the transcriptome database of Korean black raspberry, and its function was authenticated by complementary tests in Arabidopsis transparent testa 5-1 (tt5-1) mutant, which is devoid of CHI potential. Comparative transcriptomics of two strains of *Magnolia sprengeri* Pampanini, and Nuovo Giorn with red and white flowers, revealed some key enzymes of the flavonoid biosynthetic pathway, such as phenylalanine ammonia-lyase, cinnamate-4-hydroxylase, *F3′H*, *F3H* and *CHS*. The families of *MYB*, *bHLH* and *WD40* transcription factors also regulate the anthocyanidin metabolic pathway. With FPKM investigation, eight of these genes for transcription factors associated with the transcript abundance of genes, metabolic processes, and the color of flowers showed an eightfold boost in expression level in *Magnolia sprengeri* Pampanini, and Nuovo Giorn red-flowered strain, compared to the white-flowered strain [124]. In *Chlorophytum borivilianum* Santapau & R.R.Fern., which is an endangered species, an adaptogen, antiaging agent and promoter of general health, the transcriptome analysis enabled insights into the molecular mechanism of flavonoid glycosylation [125]. Transcriptome analysis in different stages of grapefruit ripening was done and the gene expression profiling was clustered by K-means grouping of RPKM [126]. To conclude, the transcriptomics of medicinal plants provided information on the relationship between genes and plant metabolites, the expression profiling of genes, key characteristics, and the molecular bases of secondary metabolite formation and biosynthetic pathways, such as iridoid, flavonoid, terpenoid, podophyllotoxin, terpenoid-derived tanshinone and the salvianolic acid secondary metabolite pathway. Transcriptomics is more viable than comparative genomics. The details of the medicinal plants reviewed are given in Table 1.

## 5. Proteomics and Functional Analysis of Proteins in Medicinal Plants

The field of proteomics is a potent platform to investigate comprehensively the proteins regulated by the drugs and explore signaling pathways of cell perturbations. Proteomics has a range of functions in medicinal plant research. Proteomics illustrates the structures, functions and modifications of proteins, and the protein–protein interactions taking place under in vitro and in vivo conditions [127]. The authentication of post-translational modifications, such as protein phosphorylation, protein acetylation, protein glycosylation and proteolysis, can also be performed [128]. These protein modifications arise during disease progression or after the treatment of a disease with drugs, or even naturally under controlled conditions. The mechanism of action of drugs is examined by the macro-investigation of alterations in proteins and by the detection of proteins that undergo modification as potential targets of the drugs [127]. The multifunctional field of proteomics helps in the prediction of the protein targets of plant-based bioactive compounds, and also provides a logical approach to appreciating the mechanisms of traditional Chinese medicine (TCM) in tumor cells and protein–drug interactions at the molecular level [129]. Terpenoids, flavonoids, glycosides and other secondary metabolites identified from TCM plants have antitumor potential in various cancers, as studied extensively via proteomics. The natural plant drug molecules cause the suppression of tumors by completely targeting the mitochondria present in the cells of malignant tissue [129]. Luteolin, Baicalein, and Tangeretin, which are the natural flavones, show signs of anticancerous activity; however, their mode of action is ambiguous. The baicalein up-regulates peroxiredoxin-6, causing reductions in reactive oxygen species (ROS) generation and hindrances in the cell proliferation of colorectal cancer [130]. The natural flavone Luteolin shows analogous anticancerous potential against various categories of cancers, together with hepatic cancer in humans. *Tripterygium wilfordii* Hook. f. has been extensively and effectively utilized for the treatment of several human syndromes, for instance rheumatoid (RH) arthritis and skin psoriasis. The anticancerous value and intrinsic mechanisms of action of *Tripterygium wilfordii* Hook. F. have been examined, and at the proteomic level the effect of a bioactive metabolite diterpenoid epoxide triptolide in curing colon cancer has been demonstrated [131]. The metabolite triptolide stimulates division at the cellular level, and a key protein 14-3-3ξ, involved in the arrest of the cell cycle and the death of cells, undergoes perinuclear translocation [132]. *Andrographis paniculata* (Burm. F) also contains diterpene compounds that have medicinal applicability against various human disorders, such as cancer and hepatitis, and viral and pathogenic bacteria [133]. Proteomic study is a proficient method for achieving a complex understanding of the inheritable traits and the physiological status of plant members of the family Acanthaceae [134]. Periplocin is extracted from the tissues of the bark and stems of *Periploca graeca* L., and can help fight cancers in the lungs and colon both in the laboratory and under natural conditions via the beta-catenin/TCF signaling pathway, by means of inducing apoptosis. With tandem mass spectrometry (TMS) and 2D gel electrophoresis, the outcome of periplocin’s action on the cell line A549 of lung cancer was studied. Western blot analysis validated and investigated the proteins subjected to modifications and protein–protein interactions [135]. Curcumin from *Curcuma longa* L. has antioxidant, antineoplastic, anti-angiogenic and anticancerous value. With proteomics, the activity of curcumin in diverse cancerous cell lines was confirmed. The proteomic investigation differentiated twelve proteins with differential expression patterns that enhance functions such as transcription, glycolysis, RNA translation, the splicing of mRNA and lipid metabolism, the synthesis of proteins, protein folding and the degradation of proteins, amino acid synthesis, and the motility of cells in the MCF-7 cell line of human breast cancer [136]. The proteins undergo differential expression in HepG2 liver cancerous cells upon treatment with Berberine. Berberine is isolated from *Coptis chinensis* Franch., and its anti-proliferative properties lead to the arrest of the cell cycle at the G0 stage of mitosis and the apoptosis of cells [137]. Gambogic acid, which is a natural xanthonoid molecule, is isolated from *Garcinia hanburyi* Hook. f. resin. It has revealed promising antitumor activity in clinical trials and hinders the growth of a range of cancer cells through multiple signaling pathways [138,139,140]. In hepatocellular carcinoma, gambogic acid possibly targets Stathmin. Over eighty anticancerous metabolites were predicted to appear in species of Garcinia. *Garcinia oblongifolia* Champ. ex Benth contains the bioactive metabolite 1, 3, 6, 7-tetrahydroxyxanthone that restricts cell proliferation in hepatocellular cancerous cells via the upregulation of p16 and 14-3-3σ [141], and 1, 3, 5-trihydroxy-13, 13-dimethyl-2H-pyran [7, 6-b] xanthone can stimulate the death of cancerous cell by the suppression of Heat Shock Protein 27 [142], which plays a crucial role. From the roots of Salvia miltiorrhiza Burge (Danshen), Tanshione ⅡA, which is phenanthrene quinine, is extracted. Tanshione ⅡA also down-regulates Heat Shock Protein 27 expression in cervical cancerous cells [127]. In another study in 2014, the treatment of primary T cell lymphoma in the central nervous system with fenugreek seeds showed an incidence of tumor regression by cancer cell destruction through cytotoxins [143].

In *Catharanthus roseus* (L.) G. Don, the systematic analysis of the proteome was undertaken via 2D polyacrylamide gel electrophoresis. Mass spectrometry identified proteins such as strictosidine synthase and tryptophan synthase, involved in alkaloid biosynthetic pathway [144]. Differential proteomic analyses of leaves, flowers, and glands of *Cannabis sativa* L. with different levels of cannabinoids were performed by two-dimensional gel electrophoresis (2D-gel), followed by mass spectrometry. The counting of resolved gel spots on 2D-gel indicated the presence of at least 800 proteins in leaves and flowers. Less than 100 proteins expressed in the flowers were characterized by mass spectrometry [145]. The first comprehensive draft map of the Cannabis proteome has given evidence for the expression of over 17,269 protein-coding regions [146]. Global proteomic profiling of the *Artemisia annua* L., and quantitative targeted sub-proteomic analysis of two chemotypes of high (HAP) and low (LAP) artemisinin content enabled identification of 13403 proteins on the basis of the genome sequence annotation database, and 182 proteins on the basis of mass spectrometry, respectively [147]. MS-based proteomics of the trichome shed light on the trichome machinery in *Artemisia annua* L. and its role in the production of artemisinin [148]. Kim et al. [149] reviewed in detail the proteomics of the Panax species with the medicinal properties of anticancer, antiaging, and protection against circulatory shock, and performed comparative proteomics of root and leaf tissues of Oriental, American and Indian ginsengs. Proteomics-based knowledge provides insights into ginseng biology. Indian ginseng (*Withania sominifera* (L.) Dunal) has major secondary metabolites of withanolides. When resolved on the 2D-gels, Indian ginseng root tissue showed 56 unique spots, whereas 22 proteins were identified by MALDI-TOF/TOF [150].

To conclude, the proteomics of medicinal plants provided information on the structure, function and post-translational modifications of proteins, protein–protein interactions, protein targets of plant-based bioactive drugs, protein–drug interactions at the molecular level, and the signaling pathways of cell perturbations. This provides insights into the mechanisms of plant-based medicines in tumor cells and a complex understanding of the inheritable traits and physiological status of plant. Proteins undergo post-transcriptional and post-translational modifications. Thus, proteomics data do not always complement transcriptomics data. Therefore, future efforts require integrated omics approaches to explore the biology of any medicinal plant [149].

**Table 1:** Omics of Medicinal Plants Reported in Review.

Medicinal Plants	Reference/s
**Genomics**	
Grapes	[56,57]
*Phalaenopsis equestris* (Schauer) Rchb.f	[58]
*Brassica napus* L.	[59]
*Capsicum annuum* L.	[60,61]
*Momordica charantia* L.	[62]
*Coffea canephora* Pierre ex A. Froehner	[63,74,75]
*Salvia miltiorrhiza* Bunge	[64]
*Ziziphus jujuba* Mill.	[65,66]
*Glycyrrhiza uralensis* Fisch. ex DC.	[67]
*Dendrobium officinale* Kimura et Migo	[68]
*Azadirachta indica* A. Juss., 1830	[69]
*Catharanthus roseus* (L.) G.Don	[45,70,72]
*Pogostemon cablin* (Blanco) Benth.	[71]
*Solanum tuberosum* L.	[76]
*Panax ginseng*	[77]
*Dendrobium officinale* Kimura et Migo	[78]
*Cypripedium macranthos* Sw. (1800)	[78]
*Ocimum sanctum* L.	[79]
*Ocimum basilicum* L.	[80]
*Papaver somniferum* L.	[42,43]
*Viscum coloratum* (Kom.) Nakai	[83]
*Sinopodophyllum hexandrum* (Royle) Ying	[84]
**Transcriptomics**	
*Podophyllum hexandrum* Royle	[95]
*Cannabis sativa* L.	[96,97]
*Chlorophytum borivilianum* Santapau & R.R.Fern.	[98,121]
*Catharanthus roseus* (L.) G. Don	[100,101,113]
*Curcuma longa* L.	[104]
*Ophiorrhiza pumila* Champ. ex Benth.	[94]
*Withania somnifera* (L.) Dunal	[106]
*Papaver somniferum* L.	[44]
*Salvia miltiorrhiza* Burge (Danshen)	[109]
Lupinus angustifolius L.	[110]
Camptotheca acuminata Decne.	[112]
Rauvolfia serpentina (L.) Benth. ex Kurz	[111]
Vaccinium macrocarpon Aiton 1789	[101]
Blueberry (Vaccinium sp.)	[115]
*Rubus coreanus* Miq. 1867	[116]
*Magnolia sprengeri* Pampanini, Nuovo Giorn	[120]
Grapefruit	[122]
**Proteomics**	
*Tripterygium wilfordii* Hook.f.	[127]
*Andrographis paniculata* (Burm. F)	[129]
*Periploca graeca* L.	[131]
*Curcuma longa* L.	[132]
*Coptis chinensis* Franch.	[133]
*Garcinia hanburyi* Hook. f.	[134,135,136]
*Garcinia oblongifolia* Champ. ex Benth	[137,138]
*Salvia miltiorrhiza* Burge (Danshen)	[123]
*Catharanthus roseus* (L.) G. Don	[140]
*Cannabis sativa* L.	[141,142]
*Artemisia annua* L.	[143,144]
*Withania sominifera* (L.) Dunal	[146]
**Metabolomics**	
*Aloe vera* (L.) Burm.f.	[151]
*Panax ginseng* C.A. Mey.,	[152]
*Panax notoginseng* (Burkill) F.H.Chen	[152]
*Panax japonicus* (T.Nees) C.A. Mey	[152]
*Persicaria minor* (Huds.) Opiz	[153,154]
Artemisia	[155]
*Pulcaria crispa* (Forssk.) Benth. ex Oliv.	[155]
*Rubus coreanus* Miq. 1867	[116]

## 6. Metabolomics in Medicinal Plants

Metabolomics is mainly significant in the plant kingdom, because of the huge quantity of metabolites (primary and secondary) produced by plants [156]. Metabolomics is a potent tool for discovering new chemical entities (NCEs) for the detection and development of drugs, by helping in the discovery and profiling of secondary metabolites in therapeutic plants, the regulation of the response of drugs, and the scrutiny of possible cytotoxic effects. High-throughput screening for the assessment of plant drugs and the detection of biomarkers for revealing human ailments [53,157], the isolation and detection of metabolites, and the fingerprinting of plant metabolites are required for the improved exploitation of therapeutic plants [158]. The metabolomics of the therapeutic plants represents a promising scientific area that assists in the detection of various drug molecules [158,159,160]. Metabolomics is used in an extensive array together with synthetic biology, medical science and Ayurveda, and is useful in predictive plant system modeling. Secondary metabolites with low therapeutic value and concentrations are not easily detected in plants. Yet, synergistic biological activities are generated due to numerous intrinsic ingredients in plants and herbal formulations. Here, metabolomics operates as a proficient approach to understand the phytochemistry of a range of medicinally active herbal ingredients [158]. The genes involved in some biosynthetic pathways formulate gene clusters in the plant genome [42,161], making gene identification easier, and this also gives an additional exhaustive approach to understanding the specialized metabolites’ evolution and their function. About 1 million metabolites exist in the whole flora [162], out of which only a few have been investigated for their metabolite biological activities and chemical constituents [163]. This has paved the way for further investigations into the classification of the metabolites of plant species that are as yet unexplored. Approximately 200,000 secondary metabolites have been investigated in plant species, several of which arose from the genome duplications that further caused the fast progression of complex characters [151,152]. The three key classes of secondary metabolites of plants, based on their structural characteristics and biosynthetic pathways, are terpenoids (~36,000) (Buckingham, 2007), alkaloids (~12,000) [153] and phenolics (~10,000) [154].

Metabolomics studies undertaken with the analytical technique of nuclear magnetic resonance spectroscopy are useful for compiling metadata in two diverse dimensions for the detection of any alterations [159]. The methods of NMR spectroscopy and multivariate investigation were utilized to assess the metabolite profile and inhibitory effects in *Aloe vera* (L.) Burm. f., an anticancer and antitumorigenic plant. The metabolome influences the genome of hepatocellular carcinoma cells by escalating the gene expressions of p53 and Bcl-2 [164]. The metabolomic characterization of *Panax ginseng* C. A. Mey., *Panax notoginseng* (Burkill) F. H. Chen and *Panax japonicus* (T. Nees) C.A.Mey. by the UPLC-QTOF-MS technique identified secondary metabolites such as chikusetsu saponin Ⅳa and ginsenosides (Rb1, Rb2, Rc, Rg2, R0) [165]. In *Persicaria minor* (Huds.) Opiz syn. *Polygonum minus* Huds, 48 compounds with via GC × GCTOF MS, 42 compounds via GC-MS, 37 volatile compounds via GC-MS investigation, and 85 flavonoids via LC-TOF were successfully identified [155,166]. Over 50% of anticancer drugs used in therapeutics today have a natural source [167,168]. The profiling of metabolites and the antitumor potential of broadly cultivated plants belonging to Compositae are reported. These species of plants portray changeable metabolite profiles. The plant Artemisia has the maximum secondary metabolite concentration, while *Pulcaria crispa* (Forssk.) Benth. ex Oliv. shows proficient in- vitro anticancerous action [169]. Mass spectrometry (MS) showed enhancement in the flavonoid and anthocyanin groups and reductions in the sucrose, fatty acids, organic acids and amino acids in *Rubus coreanus* Miq. [120]. Several therapeutically important plant metabolites, along with their medicinal properties and chemical structures, are given in Table 2. To conclude, metabolomics of medicinal plants provided information on the isolation and detection of metabolites and new chemical entities (NCEs) for the development of drugs, the fingerprinting of plant metabolites, and phytochemistry.

## 7. Conclusions and Future Prospects

Modern day omics-based technology allows extensive and nano-scale assessments of biological samples for the probing of compounds with medicinal properties. Traditional medicinal plants are very prolific in the discovery of novel plant-based drugs. Medicinal plants have unexploited potential for use in novel molecular target discovery, which adds to the process of drug development. It is imperative to manipulate and investigate not only at the genome level with high-throughput sequencing and recombinant DNA technology, but also at the levels of the proteome with mass spectrometry, the transcriptome with RNA Seq and the metabolome with nuclear magnetic resonance spectroscopy and/or LC-MS. In modern day biology, big data is derived from omics technologies, which allow the discovery of unidentified metabolic pathways/enzymes, metabolites, genes, gene networks, and protein–protein interactions, because of which interdisciplinary research on medicinal plant genomics accompanied by high-throughput sequencing platforms for DNA and RNA, metabolomics and proteomics is entirely essential. These studies will continue to disclose novel secondary metabolites that are biologically active and thus make the immense biological diversity of plants applicable for use in for novel drug discovery. The systems biology tool will help in identifying networks by which pharmacological substances of individual medicinal herbs or synergistic action networks of herbal prescriptions can be resolved and their target signaling pathway networks can be studied. However, the databases, computational models and infrastructure required to pool the disciplines are in their infancy. Systems biology will prove to be a revolutionary research area in plant-based drug discovery and diseases in future.

## Figures and Tables

**Table 2:** Medicinal Properties and Chemical Structures of Some Metabolically Bioactive Compounds from Medicinal Plant Species. (Chemical Structures were Drawn with ChemSpider Software).

Medicinal Plants	Medicinal Metabolites	Drugs/Synthetic Derivatives	Medicinal Properties	Structures	Reference/s
*Ammi visnaga*	Visnadin, visnagin, and khellin	Amiodarone for cardiac dysrhythmias; Cromolyn for treatment of asthma	Kidney stones, menstrual cramps to atherosclerosis. Cardiac arrhythmias, congestive heart failure, angina, and hypercholesterolemia	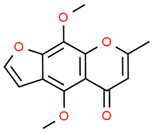 Khellin	[170,171,172]
*Andrographis paniculata* (Burm.f.) Nees	Andrographolide	Anti-inflammatory, antibacterial, antitumor, antidiabetic, antimalarial and hepatoprotective		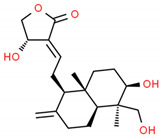	[173]
*Apium graveolens* L.	L-3-n-butylphthalide drug		Promising candidate for treatment of cerebral ischemia, Parkinson’s and Alzheimer’s disease	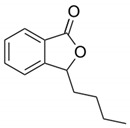	[174]
*Artemisia annua* L.	Artemisinin	Artemether, artemether and artesunate	Antimalarial and antiviral activity and resist infections from protozoans	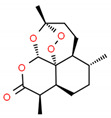	[175]
Atropa belladonna, *Datura stramonium*	Atropine/Hyoscyamine/Scopolamine	Donnatal	peripheral anticholinergic or antispasmodic action	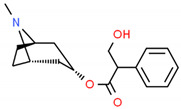	[176]
*Bacopa monniera* L.	Bacoside A3, bacopaside 1, bacopaside 2, jujubogenin, bacosaponine C		Neuromedicine for various disorders such as anxiety, depression and memory loss	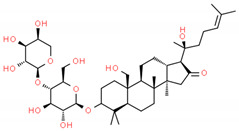	[177]
*Camptotheca acuminata* Descne and *Nothapodytes nimmoniana* (J. Grah.) D.J. Mabberley	Camptothecin	Topotecan and irinotecan	Anticancer	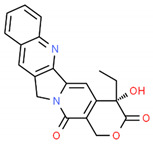	[178]
*Cannabis sativa* L.	Cannabinoids		Anti-inflammatory, anticancer, analgesic, muscle relaxant, neuro-antioxidative and psychoactive drugs	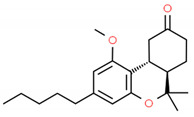	[179]
Capsicum sp.	Capsaicine	Apsaicin 8% patch (Qutenza™)	Topical analgesic, Neuropeptide-releasing agent selective for primary sensory peripheral neurons	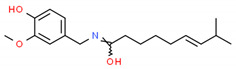	[180]
*Catharanthus roseus* L.	Vinblastine and vincristine		Anticancer drugs	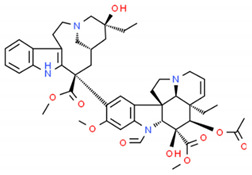 Vincristine	[181]
				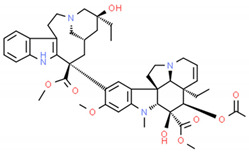 Vinblastine	
*Cinchona officinalis* L.	Natural quinine, quinidine	Chloroquine	Antimalaria	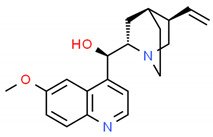	[182]
*Colchicum autumnale* L.	Colchicine	Colchicine analogs namely, 3-demethyl colchicine, colchicoside, thiocolchicocide	Anticancer	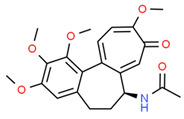	[183]
			Anti-gout and familial Mediterranean fever (FMF), pericarditis, coronary artery disease and other inflammatory and fibrotic conditions		[184]
*Curcuma longa* L.	Curcuminoids mainly curcumin		Antioxidant, neuroprotective, antitumor, anti-inflammatory, anti-acidogenic, radioprotective and anti-arthritis	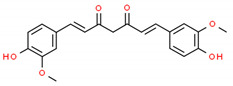	[185]
*Digitalis* *lanata*	Digoxin/digitoxin	Digitalis (digoxin) sold under brand name Lanoxin and digitoxin by brand name Crystodigin	Heart medicine	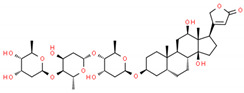 Digoxin	[186,187]
				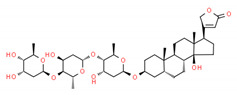 Digitoxin	
Dioscorea species	Diosgenin steroidal sapogenin		Sex hormones, corticosteroids, oral contraceptives and steroidal drugs	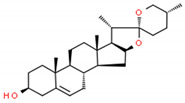	[188,189]
*Erytrhoxylum coca* Lam.	Cocaine		Topical anesthesia of the eye	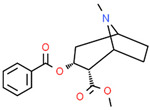	[190]
*Glycina max* (L.) Merr	Genistein		Antiosteoporosis, anti-inflammatory, anticancer, antioxidant, antidiabetic, and antiobesity activities	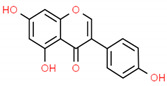	[191]
*Nicotiana tabacum* L.	Nicotine		Smoking cessation drug to relieve withdrawal symptoms	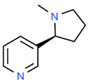	[192]
*Papaver somniferum* L.	Morphine		Musculoskeletal pain, abdominal pain, chest pain, arthritis, and even headaches	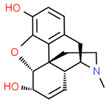	[193]
*Papaver somniferum* L.	Noscapine		Opiate analgesics, antitussive, stroke, anticancer	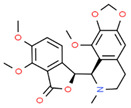 Noscapine	[194]
	Codeine		Opiate analgesics, antitussive	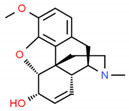 Codeine	
*Picrorhiza kurroa* Royle ex Benth.	Picroside		Picrosides as anticarcinogenic agents, hepatoprotective drug formulation, picroliv	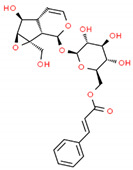	[195,196,197]
*Podophyllum hexandrum* Royle (syn. P. emodi Wall.) and Podophyllum peltatum L.	Podophyllotoxins	Etoposide and teniposide	Anticancer drugs	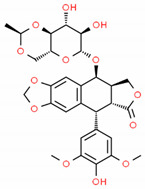	[198]
*Rauwolfia serpentine* L.	Reserpine		Hypertension	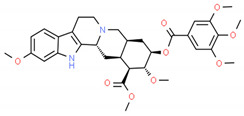	[199]
*Salvia miltiorrhiza* Burge (Danshen)	Tanshinoate B, Danshensu, Isotanshinone ⅡA and Cryptotanshinone		Small-molecule cardiovascular drug discovery	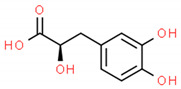 Danshensu	[200]
*Taxum brevifolia* Nutt.	Taxol	Paclitaxel		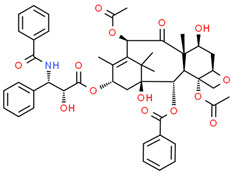	[201]
